# Maternal Mortality in Africa: Regional Trends (2000–2017)

**DOI:** 10.3390/ijerph192013146

**Published:** 2022-10-12

**Authors:** Luc Onambele, Wilfrido Ortega-Leon, Sara Guillen-Aguinaga, Maria João Forjaz, Amanuel Yoseph, Laura Guillen-Aguinaga, Rosa Alas-Brun, Alberto Arnedo-Pena, Ines Aguinaga-Ontoso, Francisco Guillen-Grima

**Affiliations:** 1 School of Health Sciences, Catholic University of Central Africa, Yaoundé 1110, Cameroon; 2 Epidemiology and Public Health Program, Department of Surgery, Medical and Social Sciences, University of Alcala de Henares, 28801 Madrid, Spain; 3 Department of Health Sciences, Public University of Navarra, 31008 Pamplona, Spain; 4 San Juan Health Center, Primary Health Care, Navarra Health Service, 31006 Pamplona, Spain; 5 National Epidemiology Centre, Carlos III Health Institute, 28029 Madrid, Spain; 6 REDISSEC and REDIAPP, 28029 Madrid, Spain; 7 School of Public Health, College of Medicine and Health Sciences, Hawassa University, Hawassa P.O. Box 5, Ethiopia; 8 Department of Nursing, Clínica Universidad de Navarra, 31008 Pamplona, Spain; 9 Epidemiology Division, Public Health Center, 12003 Castelló de la Plana, Spain; 10 Public Health and Epidemiology (CIBERESP), Instituto de Salud Carlos III, 28029 Madrid, Spain; 11 Department of Preventive Medicine, Clínica Universidad de Navarra, 31008 Pamplona, Spain; 12 Facultad de Ciencias de la Salud, Universidad Pública de Navarra (UPNA), Avda. de Baranain sn, 31008 Pamplona, Spain; 13 Instituto de Investigación Sanitaria de Navarra (IdiSNA), 31008 Pamplona, Spain; 14 Center for Biomedical Research Network, Physiopathology of Obesity and CIBER-OBN, Instituto de Salud Carlos III, 28029 Madrid, Spain

**Keywords:** Africa, maternal mortality rate, joinpoint regression analysis, mortality, trends

## Abstract

Background: United Nations Sustainable Development Goals state that by 2030, the global maternal mortality rate (MMR) should be lower than 70 per 100,000 live births. MMR is still one of Africa’s leading causes of death among women. The leading causes of maternal mortality in Africa are hemorrhage and eclampsia. This research aims to study regional trends in maternal mortality (MM) in Africa. Methods: We extracted data for maternal mortality rates per 100,000 births from the United Nations Children’s Fund (UNICEF) databank from 2000 to 2017, 2017 being the last date available. Joinpoint regression was used to study the trends and estimate the annual percent change (APC). Results: Maternal mortality has decreased in Africa over the study period by an average APC of −3.0% (95% CI −2.9; −3,2%). All regions showed significant downward trends, with the greatest decreases in the South. Only the North African region is close to the United Nations’ sustainable development goals for Maternal mortality. The remaining Sub-Saharan African regions are still far from achieving the goals. Conclusions: Maternal mortality has decreased in Africa, especially in the South African region. The only region close to the United Nations’ target is the North African region. The remaining Sub-Saharan African regions are still far from achieving the goals. The West African region needs more extraordinary efforts to achieve the goals of the United Nations. Policies should ensure that all pregnant women have antenatal visits and give birth in a health facility staffed by specialized personnel.

## 1. Introduction

The International Classification of Diseases Tenth Revision (ICD-10) from the World Health Organization (WHO) defines maternal death as “a death in a woman from any cause related to or aggravated by the pregnancy or its management (excluding accidental or incidental causes) during pregnancy and childbirth or within 42 days of termination of pregnancy, irrespective of the duration and site of the pregnancy” [1,2]. United Nations Sustainable Development Goals state that by 2030, the global maternal mortality rate (MMR) should be lower than 70 per 100,000 live births [3,4]. No country should have an MMR higher than 140 per 100,000 live births. From 2000 to 2017, the MMR decreased by 38% in the world [5,6]. In Sub-Saharan Africa, there was a reduction in MMR of nearly 40% in the same period [7].

Nevertheless, MMR is still one of the leading causes of death among African women. Socioeconomic status, parity, and living in rural areas influence maternal mortality (MM) [8,9]. Cross-national or civil wars, insurgencies, and political upheavals also significantly influence maternal and infant mortality [10,11,12,13,14]. The same goes for economic crises, famines, epidemics, and subsistence crises. The other influencing factors are the percentage of births occurring in health facilities, the proportion of pregnant women with antenatal visits, and the percentage of births attended by health personnel [15,16]. In 2012, the World Health Organization launched the Maternal Death Surveillance and Response (MDSR) policy. Many African countries participated [17,18]. There were some difficulties with maternal death surveillance and response implementation in several African countries [19,20,21,22] due to several causes, including communication problems at district or community levels, recommendations that were poorly addressed, highly political issues, low accountability, and organizational problems. Estimating maternal mortality is very difficult, especially if nobody knew about the deceased woman’s pregnancy or if the delivery was outside a health facility or attended by unskilled people who could not identify the correct cause. Several methods are used in Africa to estimate maternal mortality: demographic surveillance studies; health record review studies; confidential inquiries into maternal deaths and maternal death surveillance and response systems; prospective cohort studies; reproductive-age mortality surveys (RAMOS); direct or indirect sisterhood methods; mixed methods; and mathematical modeling. Maternal mortality ratios produced by sisterhood method studies and RAMOS studies that combined institutional records and community data are more compatible with the estimations of international organizations [23].

This study aims to compare the evolution of maternal mortality rates in different regions of Africa. This study is the first to analyze maternal mortality rates in the African Union using the joinpoint regression technique. The specific contribution of this study is that it provides information about trends and changes in them. This information is helpful for regional projections and studying the effects of external factors such as economic crises, civil wars, or health reforms on maternal mortality.

## 2. Literature Review

This review attempts to review the previous efforts of researchers on maternal mortality in Africa. This section succinctly reviews the studies available in the literature. 

Sub-Saharan Africa and South Asia have the world’s highest maternal mortality rates, accounting for 85 percent of maternal deaths worldwide. Maternal deaths are only the more visible aspect of maternal health. The morbidity and its consequences are significant. In addition to mortality, women with complications associated with pregnancy or childbirth may continue to experience long-term problems [24].

The question of how health spending affects maternal mortality is controversial. A study in South Asia showed that healthcare spending affects life expectancy, including a reduction in maternal mortality, with spending above 6% of GDP being necessary to be cost-effective [25]. Another study conducted in South Asia between 2000 and 2017 showed that increased healthcare spending increased maternal mortality rates by between 0.16% and 1.95% depending on the model used [26]. This study showed that increased GDP, access to sanitation, and clean fuel technologies were essential in reducing maternal mortality.

A study with data from 46 countries in Sub-Saharan Africa during the period 2000–2015 showed that an increase of 1% in health expenditure per capita reduced maternal mortality by 0.35% [27]. GDP growth and foreign aid were the most significant influence on health spending [28]. In Africa, maternal mortality is affected by structural socioeconomic factors such as socioeconomic instability, famine, migration, and weak health infrastructure. 

Another factor is wars and civil wars that destroy infrastructure and forced migration. Wars and civil wars can affect the provision of health services and the population’s living conditions by increasing maternal mortality [29]. In 2008, 50% of maternal deaths happened in eight countries with ongoing or recent wars, and 75% of countries with a high maternal mortality rate worldwide were at war [30]. Between 2000 and 2017, more than 25% of African countries (14 countries) have been in war or civil war at some point [31,32,33]. There were five countries in East Africa (Eritrea, Ethiopia, Rwanda, Somalia, South Sudan, Sudan), three in Central Africa (Burundi, Central African Republic, Democratic Republic of Congo), two in West Africa (Liberia and Sierra Leone), two in South Africa (Angola, Mozambique) and one in North Africa (Libya). Terrorism and war in the adjacent countries have a spillover impact on maternal mortality elevation at the regional scale [34].

Almost 31% of maternal deaths happen during pregnancy, 36% at delivery or in the first week, and 33% from 1 week to 1 year [35]. In the Global Burden of Disease Study, the leading causes of maternal death were hemorrhage and hypertensive diseases [36]. The same was found in literature reviews [37]. Maternal mortality causes vary, including maternal hemorrhage, sepsis and pregnancy infections, hypertensive disorders of pregnancy, obstructed labor, abortion, HIV, other maternal disorders, and late maternal deaths [38]. The leading causes of adolescent MM are postpartum hemorrhage [39,40], hypertensive disease, and puerperal sepsis [40]. Hemorrhage is linked with delivery assisted by unskilled people, at home or in ill-equipped primary healthcare centers, and with the absence of consumables or access to transfusion [39,41,42]. There are also delays in seeking health care and evacuation to a hospital in complicated cases due to cultural seeking behaviors, long distances, and the problem of transportation [43,44,45]. One useful model is that of the “three delays” [46]. Delays can be classified into three categories: delay in seeking health care for an obstetric emergency; reaching a health facility; and being assisted once the obstetric facility is reached. The creation of maternity waiting homes for pregnant women annexed to health centers and hospitals can reduce maternal and hospital mortality [47].

One meta-analysis in Sub-Saharan Africa found that the leading causes of mortality were obstetric hemorrhage followed by hypertensive disorders in pregnancy, non-obstetric complications, and pregnancy-related infections. The leading cause of the hemorrhage group was postpartum hemorrhage [41].

One proposal has been the so-called Service Delivery Redesign for Maternal and Newborn Health, which consists of moving deliveries from primary care health centers to obstetric hospitals or “delivery hubs” prepared to attend cesarean sections and provide transfusions [48]. However, this measure has not yet been evaluated [49].

Pre-eclampsia/eclampsia was the leading cause in the hypertensive group, and puerperal sepsis was the leading cause in the pregnancy-related infections group [41]. Antecedents of postpartum hemorrhage and multiparity are risk factors for postpartum hemorrhage. Prenatal visits are essential for detecting women at high risk [50]. 

Before 2016, WHO recommended at least four prenatal visits, one in the first trimester [51]. The World Health Organization now recommends that pregnant women have at least eight visits during pregnancy. Visits during pregnancy prevent complications and facilitate early detection and treatment of complications contributing to a healthy pregnancy [52,53]. 

Non-obstetric causes of mortality play an important role in maternal mortality in Africa, among them malaria, tuberculosis, and HIV. Malaria is the first non-obstetric cause of MM and can explain regional differences. In malaria-endemic areas, Plasmodium falciparum prevalence is high in young women because of the uncommon use of insecticide-treated nets before their first pregnancy. The prevalence of malaria at the first prenatal visit is influenced by season and country. During the rainy season, prevalence was 59.7% in Ghana, 56.7% in Burkina Faso, 42.2% in Mali, and 16.8% in Gambia; meanwhile, in the same countries in the dry season the prevalence was 41.3%, 34.4%, 11.5%, and 7.8% [54]. Tuberculosis and HIV are highly prevalent among pregnant women in Sub-Saharan Africa [55].

## 3. Materials and Methods

### 3.1. Region Classification

**Figure 1:**
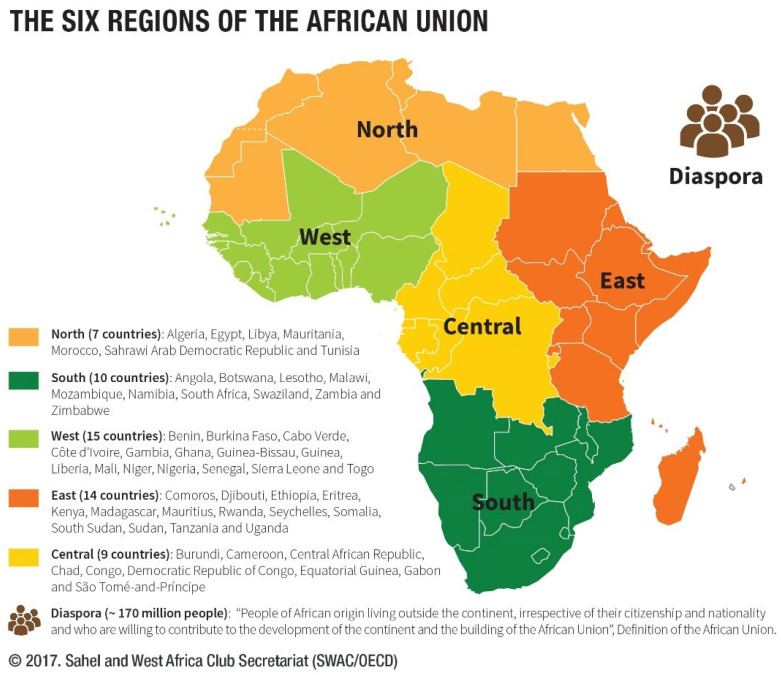
Regions of the African Union, according to the African Union classification.

We used the five regions of the African Union (Figure 1): North (Algeria, Egypt, Libya, Mauritania, Morocco, Tunisia); East (Comoros, Djibouti, Eritrea, Ethiopia, Kenya, Madagascar, Mauritius, Rwanda, Seychelles, Somalia, South Sudan, Sudan, Tanzania, Uganda); Central (Burundi, Cameroon, Central African Republic, Chad, Congo, DR Congo, Equatorial Guinea, Gabon, São Tomé and Príncipe); South (Angola, Botswana, Lesotho, Malawi, Mozambique, Namibia, Eswatini, South Africa, Zambia, Zimbabwe); and West (Benin, Burkina Faso, Cabo Verde, Côte d’Ivoire, Gambia, Ghana, Guinea-Bissau, Guinea, Liberia, Mali, Niger, Nigeria, Senegal, Sierra Leone, Togo). The African Union’s classification of regions includes a sixth region of “peoples of African origin living outside the continent, irrespective of their citizenship and nationality…” [56,57]. This sixth region has not been considered in the analysis. 

### 3.2. Data Sources

MMRs and population data were extracted from UNICEF mortality databases from 2000 to 2017 (2017 is the last date, updated in 2021) [58]. The annual mortality rates for each region and Africa were estimated by weighting each country’s MMR with its population [59]. We extracted the mother’s age at the first birth in African countries from an international database [60]. We extracted the number of prenatal visits from the DHS and UNICEF databases and the proportion of deliveries in health facilities [61].

The Western Sahara and British and French territories were excluded. Western Sahara was not included because its MMR is not included in the UNICEF database. British and French territories were excluded because they are not African Union members.

### 3.3. Joinpoint Regression

Joinpoint regression has been widely used in the study of chronic diseases to detect periods of sustained changes in the incidence rates. Joinpoint regression was performed to detect changes in the trends. To describe the magnitude of the change in each trend, we estimated the annual percentage change (APC) and calculated the 95% confidence intervals (95% CI). 

### 3.4. Study Variables

The MMR was the dependent variable in these models, and the year of death was the independent variable. 

### 3.5. Autocorrelation Empirical Models

The existence of autocorrelation in the time series was estimated using the Durbin–Watson test [62,63,64]. There was a high positive autocorrelation (Durbin–Watson test = 0.252). The models were first fitted with the uncorrelated errors option. Subsequently, the analysis was repeated, considering the autocorrelation parameter. As there were substantial differences between the two models, the model with autocorrelation was chosen. 

### 3.6. Comparison between Regions

We compared the regional rates using a parametric ANOVA and a distribution-free Kruskal–Wallis. We performed a post hoc contrast with Tamhane T2 and a Pairwise Wilcoxon Rank Sum Test with corrections for multiple testing. In all analyses, *p* values < 0.05 were considered statistically significant.

Comparability tests were performed to compare two sets of trend data whose mean functions were represented by joinpoint regression with pairs of regional trends. Specifically, we computed coincidence tests to evaluate if two joinpoint regression functions were identical and tests of parallelism to evaluate whether the two regression mean functions were parallel [65]. 

### 3.7. Software

Computations were completed with Minitab version 17 [66], IBM SPSS v.22 [67], RStudio 2022.02.3 [68,69], and Joinpoint Regression [70,71].

## 4. Results

### 4.1. Analysis of the African Continent

**Figure 2:**
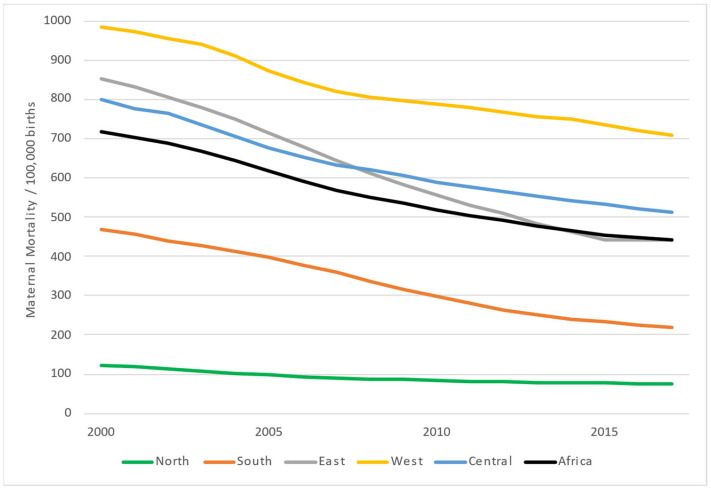
Evolution of maternal mortality rates in Africa (2000–2017).

In Figure 2, the evolution of the regional MMRs is shown. The difference between the regions with the highest and the lowest MMR decreased from 860 per 100,000 births in 2000 to 633 in 2017. In other words, interregional inequalities decreased 26% with time. 

**Figure 3:**
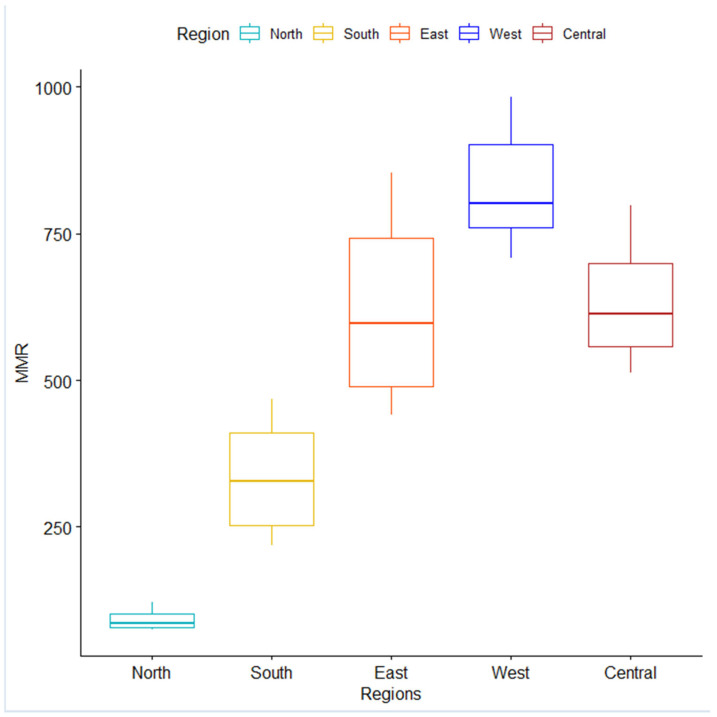
Maternal Mortality Rates in Africa (2000–2017) by region.

Figure 3 shows a boxplot for the annual regional rates from 2000–2017. East Africa showed the highest variability among the five regions, and North Africa showed the lowest. Each region displayed a relatively symmetric distribution of its MMR. The West African region showed the highest MMR, and North Africa showed the lowest.

We conducted a parametric one-way ANOVA and a distribution-free Kruskal–Wallis’s test for the MMR that found significant differences in MMR among regions (*p* < 0.001). We conducted a post hoc analysis and found that Tamhane’s T2 and the pairwise Wilcoxon contrasts showed differences between all paired regions. The only exception was Central and East Africa, where no difference was detected. Despite the results of the post hoc contrasts, the comparison of the trends of the East African and Central African regions was non-coincident (*p* < 0.001) and non-parallel (*p* < 0.001).

**Figure 4:**
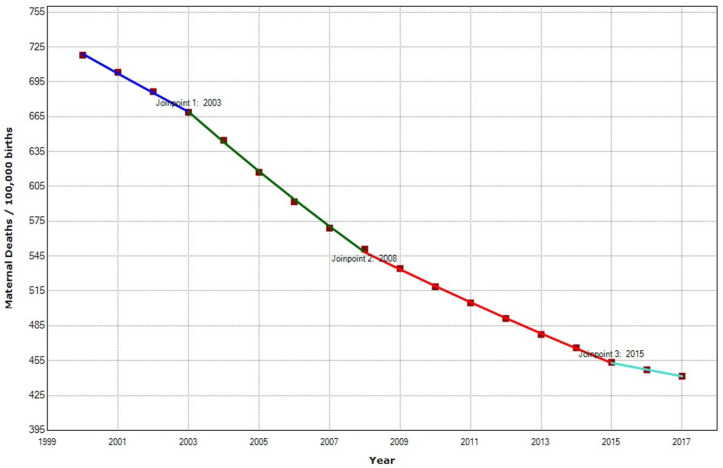
Maternal Mortality trends in Africa (2000–2017) indicate joinpoints at the transitions between colored lines.

MMR has significantly declined in Africa, from 718 maternal deaths per 100,000 live births in 2000 to 442 in 2017 (Figure 4). However, 205,670 women still died in Africa in 2015. Most maternal deaths (203,000) occurred in Sub-Saharan Africa [72]. Three joint points have been detected, 2003, 2008, and 2015, which define four periods in which there was a substantial change in maternal mortality trend.

MMR declined from 718 deaths per 100,000 births in 2000 to 442 deaths per 100,000 births in 2017, a decrease of 38.5%. Table 1 shows that there was a substantial decline in MMR by a significant APC (−3%) (*p* < 0.001). From 2000–2003, there was a moderate decrease with a −2.4% APC, followed by a higher reduction in maternal mortality with an APC of −3.9%. The accelerated trend was interrupted in 2008 when there was a slowdown in the APC. APC changed from −3.9% in the 2003–2008 period to −2.7% in 2008–2015 and −1.3 in 2015–2017.

### 4.2. Analysis of African Regions

The North and South African regions consistently retained a lower MMR throughout the period. There was a convergence between the Central and East African region. Finally, the West African region, which started with very high levels, experienced a sharp decline but remained high (Figure 2).

**Figure 5:**
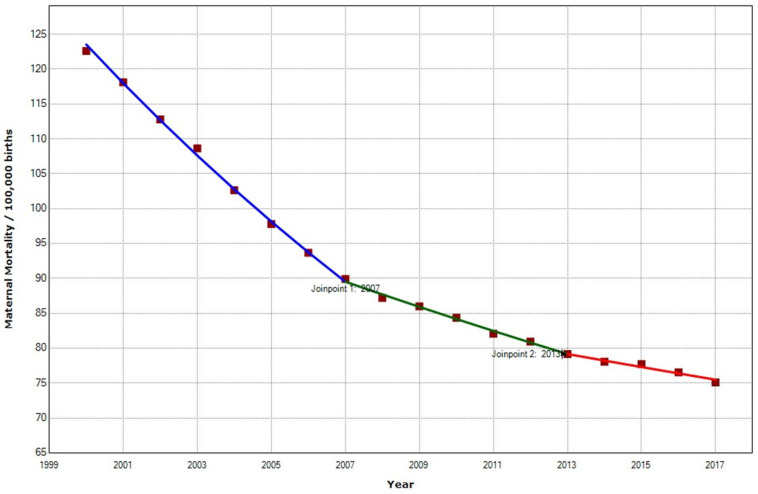
Maternal mortality trends in North Africa (2000–2017) indicate joinpoints at the transitions between colored lines.

In the North African region, the overall MMR decreased 38.74% from 122.58 to 75.09 maternal deaths per 100,000 births. A statistically significant decrease of −2.8% (95% CI −3.5; −2.2) in the MMR, with two joinpoints in 2007 and 2013, was found (Table 2, Figure 5).

**Figure 6:**
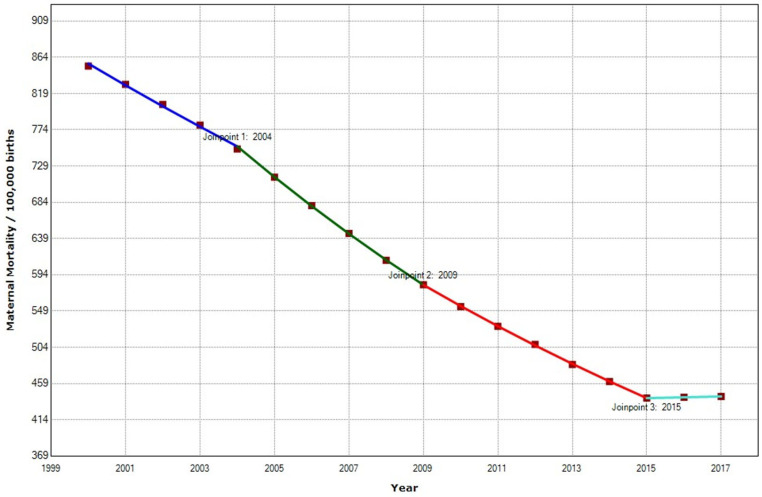
Maternal mortality trends in East Africa (2000–2017) indicate joinpoints at the transitions between colored lines.

In the East African region, the overall MMR decreased from 853 to 443 maternal death per 100,000 births (Table 2). East Africa was the region with the second highest decrease during the study period. MMR was reduced by −48,07%. Maternal mortality in the region decreased annually by 4.3%, with three joinpoints in 2004, 2009, and 2015 (Figure 6).

**Figure 7:**
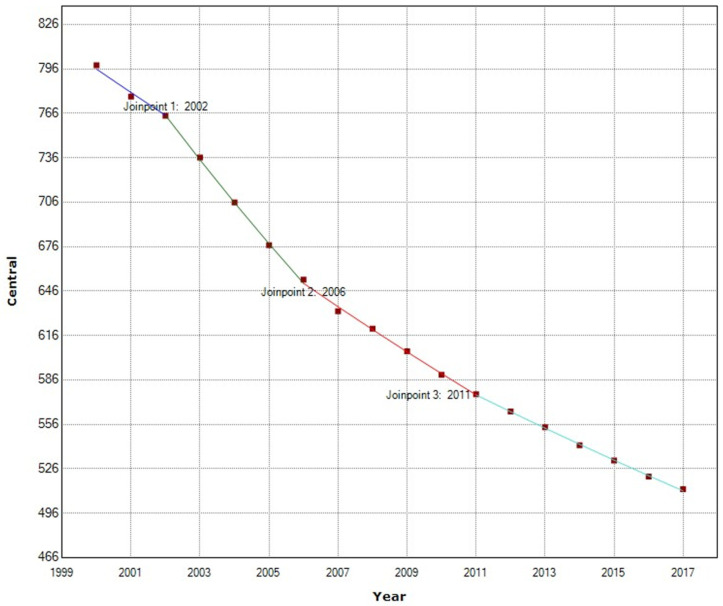
Maternal mortality trends in Central Africa (2000–2017) indicate joinpoints at the transitions between colored lines.

In the Central African region, during the period 2000–2017, maternal mortality decreased by 35.84%, from an MMR of 798.35 in 2000 to 512.19 in 2017. The APC during the whole period was −2.7% per year (95% IC −2.8; −2.5). We detected three joinpoints in 2002, 2006, and 2011. Since 2006, the APC has been progressively decreasing, first to −2.4% in 2006–2011 and later to −2.0% in 2011–2014 (Figure 7).

**Figure 8:**
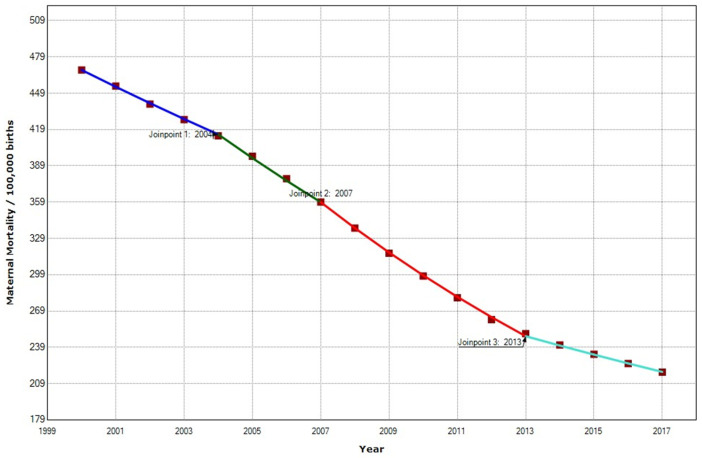
Maternal mortality trends in the South African region (2000–2017) indicate joinpoints at the transitions between colored lines.

In the South African region, the overall MMR decreased from 468 to 218 per 100,000 births. The South African region has the highest MMR reduction, with a reduction of −53.42%. We recorded a statistically significant annual decrease of −4.8% in the MMR, with three joinpoints in 2004, 2007, and 2013 (Figure 8). In the first period, MMR decreased with an APC of −3.0%, then in the second period, 2004–2007, MMR decreased with an APC of −4.7%. Later, from 2007–2013, there was a high APC decrease of −6.0. This decrease is the greatest detected in this study. In 2013–2017, there was a deceleration with an APC of −3.1%.

**Figure 9:**
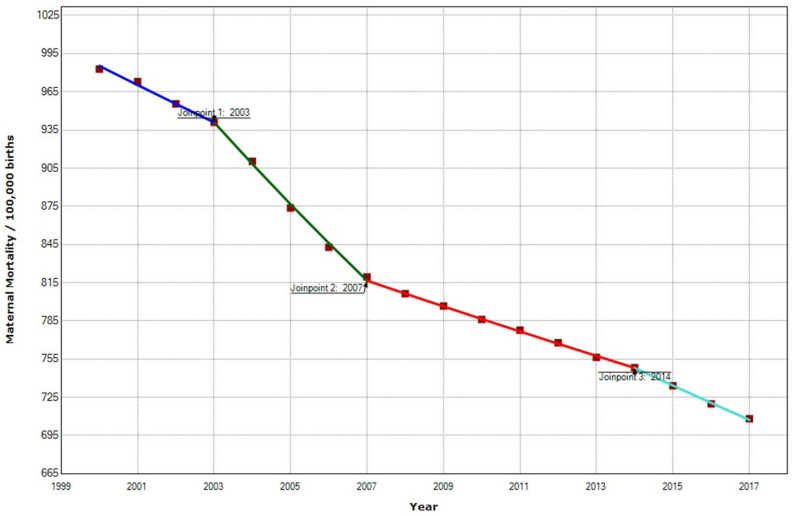
Maternal mortality trends in the West African region (2000–2017) indicate joinpoints at the transitions between colored lines.

In the West African region, the overall MMR decreased 27.90% from 982 to 708 maternal deaths/100,000 births during 2000–2017. We recorded a statistically significant decrease in APC of −2.0% in the MMR during the whole period, with three join points in 2003, 2007, and 2014 (Figure 9). 

## 5. Discussion

We detected a slowdown in maternal mortality reduction in Africa since the economic crisis that began in 2007. The decrease was detected in the North, Central, and West African regions. The East African region was somewhat affected, while the South African region was not affected by the economic crisis. The United Nations set out, in 2015 within the Sustainable Development Goals, to reduce MMR to 70 per 100,000 live births by 2030. In 2015, the global rate in Africa was 459 deaths per 100,000 population. In OCDE countries, the mean MMR in 2017 was 10.18 (95% CI 5.61–14.76) [58].

Maternal mortality in the North African region had a value in 2017 of 75 per 100,000 births, which is within the range (2–83) of the Organisation for Economic Co-operation and Development (OECD) countries (Table 3). The other African regions are very far away from the OECD countries.

Although Africa has experienced a considerable reduction in its MMR of 718 deaths per 100,000 population in 2000, there is still a long way to go to reach the target. MMR rates would have to fall in Africa with an APC of −15.29% to reach the target in 2030. This reduction is a figure that has never been achieved. If we continue with the APC of 2000–2017 (−3.0%), the target will be reached in 2086.

The only exception is North Africa, the most advanced region, where the proposed target should be reached around 2023.

Although this may seem disheartening, there are signs of hope. A commitment is needed from the health authorities to deploy a health policy that allows easy access to health services for all pregnant women. An example would be Ethiopia’s case, where the MMR decreased from 871 per 100,000 in 2000 to 412 per 100,000 in 2017. The MMR is still far from the target rate of 70, but it represents a reduction of more than 50% [73].

There has been a steady decline in maternal mortality in the South African region. The decline has been remarkably resilient as it was not affected by the economic crisis that hit the South African region from 2008 onwards. Changes in health policy and legislation may be responsible for these declines despite adverse economic conditions [74].

There are differences in mortality causes between regions. Although obstetric hemorrhage is the leading cause of maternal death, pregnancy infection is the fourth in all the regions of Sub-Saharan Africa. The second cause is hypertensive disorders in pregnancy in the West and East African regions, while in South Africa, the second cause is non-obstetric complications [41].

In the North African region, maternal mortality has been declining. Nevertheless, there was a deceleration in the APC that moved from −4.5% in 2000–2007 to −2.0% in 2007–2013. Two factors may have affected maternal mortality during this period: the 2007 economic crisis [75,76,77] and the Arab Spring (2010–2011) [78,79,80,81,82,83,84].

We have detected a slowdown in the APC coinciding with the 2007 economic crisis in the North, Central, and West African regions. The most significant impact was in the West and North African regions, where the APC decreased by 65.71% and 13%, respectively. The East African region was not affected by the economic crisis. In the South African region, there was an acceleration in the APC. The GDP increase in the region between 2008 and 2011 could explain this [85,86,87,88].

**Figure 10:**
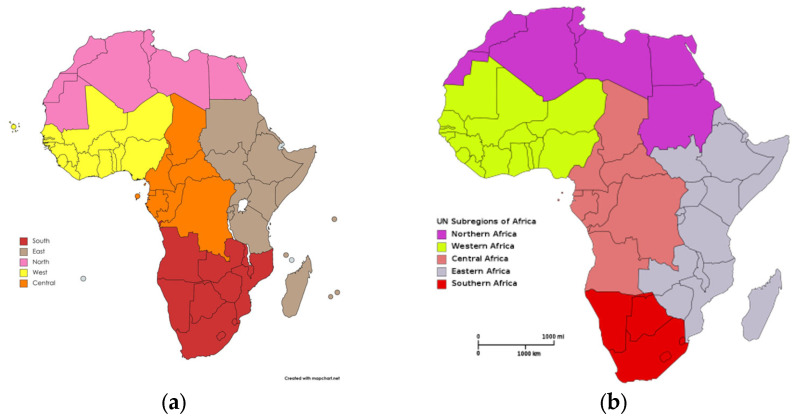
African Regions Classifications (**a**) African Union; (**b**) United Nations, Source: CC BY-SA 3.0, Available online: https://commons.wikimedia.org/w/index.php?curid=546265 (accessed on 14 July 2022).

A debatable question is which classification to use for the regions of Africa. Many international organizations use the United Nations Region Classification of the M49 Standard, classifying African countries into five regions (Figure 10) [89,90].

The UN classification includes Sudan in Northern Africa [91]. Likewise, the South African region is much larger in the African Union classification because it includes Angola, Zimbabwe, Zambia, and Mozambique (Figure 10).

Choosing one or another classification of the regions has implications because it affects regional mortality rates. Our study used the African Union classification instead of the United Nations Classification, which we decided to use because our data could be more beneficial for elaborating regional policies within the African Union.

In many countries, mortality data collection systems are not very comprehensive, which is a limitation of the study. There are various methods for the estimation of maternal mortality. Estimates from international agencies may differ from official national statistics. Indeed, a study in Ethiopia showed that maternal mortality data provided by international agencies underestimated maternal mortality: 401 versus 412 per 100,000 [92]. Death registration and recording of the cause of death as a part of the vital statistics system are deficient in many countries of Africa, and in 2015 the regional average completeness rate of death registration was only 34.6% [93]. In addition, some countries have reported low death registration, and few countries have achieved international standards in this respect [94]. In some African countries, maternal deaths have been underreported [95,96].

During 2015–2020 in Sub-Saharan African countries, the principal causes of MM were obstetric hemorrhage (28.9%), hypertensive disorders (22.1%), non-obstetric complications (18.8%), and pregnancy-related infections (11.5%). 

Considering the regions of Africa, the distribution of MM causes of death was the following: Obstetric hemorrhage was the first cause in all the regions, from 25.2% (Southern) to 31.3% (Western). 

Hypertensive disorders were the second cause in the East, Central (27.2%), and West (22.7%) African regions and third in the South African region (17.8%). Non-obstetric complications were the second cause in the South African region (22.9%) and third in the East and Central (15.3%) and West (14.4%) African regions [41]. 

In the South African region, indirect maternal death from medical and surgical diseases is the fourth cause of maternal death (16.9%). Cardiac diseases cause one-third of these deaths. These diseases should be diagnosed early during prenatal visits [97].

Pregnancy-related infections were fourth in the West (13.8%), East- Central (11.8%) and South (8.8%) regions [41]. In Sub-Saharan Africa, multiple pregnancies, sickle cell disease, pregnancies at the extremes of reproductive age, and pre-existing vasculitis are risk factors for eclampsia [98].

The demographic, socioeconomic, and the geographic diversity of countries in each regional group could be considered in the explication of the results (Table 4). There may be many reasons for regional differences in mortality, one of which may be differences in maternal age. There were regional differences in maternal age at first birth. The medians of maternal age at first birth were higher in the North and South African regions (22.9 years and 20 years) than in the Central, East, and West African regions (19.6, 19.4, and 19.5 years). The two areas with lower maternal mortality are precisely those in which the mother’s age at first birth is highest. Maternal age higher than 35 years and high parity are risk factors in the West African region [99].

A systematic review performed in Sub-Saharan Africa found that the older maternal age increased attendance to between at least one and four prenatal care visits [105], although in some countries, it was found that very young first-time mothers sought prenatal care earlier. 

Our study indicates that wars and economic crises may have a short-term effect on maternal mortality in Africa. However, in some countries, post-war healthcare system reforms have decreased mortality in Africa’s South and East African regions. Five countries (Angola, Eritrea, Ethiopia, Mozambique, and Rwanda) reorganized their health services after the wars and significantly reduced maternal mortality. The typical features of health system reform in these countries were as follows: One aspect was decentralizing the health system to the regions or provinces. Another aspect was capacity building, increasing the number of health personnel (importing doctors from other countries such as Cuba, encouraging the deployment of community healthcare workers, nurses, and midwives). The state funding of the healthcare system to facilitate access to hospitals for women in labor and prenatal care was also important. Finally, another measure was improving health care quality [106]. In West Africa in Liberia, after the civil war, there was also an increase in the proportion of women with four prenatal visits and the proportion of deliveries in health services’ facilities [16]. 

Several studies on maternal mortality in Africa and other countries have indicated the importance of the political and economic context on maternal mortality, including external and internal conflicts such as wars and civil disorders [34]. The factors which decreased MM were the Gross Domestic Product and natural resource rents by increased healthcare attention. Urbanization and conflicts increased MM. In addition, a better governance commitment in each country apart from the level of wealth is associated with lower MM [107]. On the other hand, sub-national variations in MM need to be considered beyond national figures to explain its determinants [108]. An example could be the situation of the Maghreb, with MM rates close to United Nations targets required but with high regional disparity and high differences in MM rates [109]. 

Maternal mortality has declined in Africa, especially in southern and high-income countries. The most important long-term factors are health policies, the accessibility of health services, and the ability to sustain follow-up during pregnancy and postpartum. Accessibility is influenced by the quality of care and road building construction that facilitates transportation to health facilities [110,111]. There are data on pregnancy follow-up, but not in all African countries. The number of prenatal visits during pregnancy is low. The proportion of pregnant women with four or more visits ranges from 31.4% in Chad to 91.8% in Ghana, with a median of 59.5% [61,100,101,102]. There are differences among regions. The North African region has a higher proportion of mothers, with 72.3% of women with four or more prenatal visits, while the Central region has a lower proportion, with 50.8% (Table 4).

In Africa, 62.2% of deliveries occur in health facilities (Table 4). There are differences among regions. The highest proportion of deliveries in health facilities is in the North African region (84%), while the lower regions are the East and West African ones, with 51.2% and 51.4% deliveries at health institutions (Table 4). In the West and Central African regions, the proportion of women receiving care from a professional at birth is also low [112]. Another problem in South and East Africa is the delay in accessing healthcare. Delays in receiving adequate care once reaching a health facility, deciding to seek care in an obstetric emergency, and reaching an appropriate obstetric facility are responsible for 32–36.3%, 33–36%, and 27.6–29% of maternal deaths, respectively [113,114].

Treatment during pregnancy in HIV-exposed women with antiretroviral medication (ARV) in Africa can be used as a proxy [115]. There are variations between regions, with the West African region having the highest accessibility to the health system during pregnancy with 97.9%, while the South African region has the lowest figure of 80.2% (Table 5). Care sustainability during pregnancy is lower in South Africa (71.9%). West Africa has the lowest puerperium sustainability (17.1%). All these differences in the capacity to access the health system and use resources may influence maternal mortality.

The lack of recourses to maternal health care and the acceptability and affordability of maternal health services are factors in MM in Sub-Saharan countries [112]. Nevertheless, the main problem in Sub-Saharan Africa is not so much the accessibility of health services but that women seek care late in pregnancy. In Uganda, almost all women (97.5%) seek prenatal care during pregnancy, but only 30% during the first trimester and only 60% have four or more visits [116]. A study in Rwanda showed that mothers were the group that had the most significant influence on the decision of pregnant women to seek care [117]. In Africa, mobile phones are displacing radio and television as a means of social communication [118]. Mass media and educational interventions based on SMS and voice message reminders should be used to increase pregnant women’s prenatal visits [119,120].

One study in the South African region found that approximately one-third of maternal deaths occurred outside health facilities [121]. Long distances to health centers and hospitals could explain deaths outside health facilities. All pregnant women in Africa should have access to obstetric care that provides a safety net in an obstetric emergency. Contributing factors are the problem of transportation and health-seeking behaviors [43,44,45].

In the Sub-Saharan region, hemorrhage and eclampsia cause 40% of maternal deaths [122]. Young maternal age and multiparity are risk factors for primary postpartum hemorrhage in the West African region [123]. The lack of transfusions available in hospitals in West Africa may contribute to hemorrhages [42]. Puerperal sepsis is a significant cause of death due to the lack of sanitation and clean delivery rooms [124]. In the South African region, cesarean deliveries were the cause of puerperal infections [121]. One contributing factor was limited access to antibiotics [125]. Community health centers and referral hospitals must have trained and motivated healthcare providers [126], access to equipment, essential drugs, and blood transfusions delivered by experienced staff at all hours [23].

Anemia, food insecurity, no formal education, and no antimalarials during pregnancy affect maternal mortality in East African women aged 15–19 [127,128]. In the South African region, maternal deaths are associated with poor nutrition, low socioeconomic status, and lack of access to health-care facilities [129].

Due to the importance of deaths in pregnant women from non-obstetric causes, mainly infectious diseases such as malaria, tuberculosis, and HIV, screening for these diseases should be included in prenatal care visits [55,130].

In East Africa, among the factors associated with maternal mortality were non-membership in social movements intended to improve maternal and child health, low involvement of partners, medical illness, and no utilization of family planning services [131]. Women’s empowerment is associated with a reduction in maternal mortality but with low utilization of health services [119].

Preventive measures should focus on the prevention of hemorrhage. The most important thing is to facilitate deliveries in health facilities with the necessary materials and human resources. Another objective should be to control hypertension by increasing the number of prenatal visits where hypertension can be detected early and monitoring for communicable diseases such as malaria, tuberculosis, and HIV. Other structural measures, such as decentralizing health services and community actions, can help reduce maternal mortality.

Our results could serve as a reference for developing health policies in the regions with higher maternal mortality.

In future research, maternal mortality may be adjusted for potential risk factors, including income and education level, comorbidities, medical assistance, and environmental sanitation, to estimate maternal mortality trends [39,41,42,43].

## 6. Conclusions

Over the study period, maternal mortality has decreased in Africa by an average APC of −3%. All regions showed significant downward trends, with the sharpest decreases in the South African region. Only the North African region is close to the United Nations’ sustainable development goals for maternal mortality. The remaining Sub-Saharan African regions are still far from achieving the goals. These results show the need to develop regional policies to further decrease maternal mortality in Africa. Health policies should focus on ensuring that all women deliver their babies in a health facility to avoid deaths from postpartum hemorrhage. Health policies should also aim to ensure that pregnant women have at least eight prenatal visits, which would help reduce deaths from eclampsia.

## Figures and Tables

**Table 1:** Joinpoint analysis for maternal mortality rates in Africa, 2000–2017.

Periods	Years	APC (95% CI)	*p*
Total Period	2000–2017	−3.0 (−3.3; −2.59)	<0.001
Period 1	2000–2003	−2.4 (−2.6; −2.1)	<0.001
Period 2	2003–2008	−3.9 (−4.1; −4.7)	<0.001
Period 3	2008–2015	−2.7 (−2.6; −2.8)	<0.001
Period 4	2015–2017	−1.3 (−1.8; −0.8)	0.001

**Table 2:** Joinpoint analysis for regional maternal mortality rates in Africa, 2000–2017.

Periods	Years	APC (95% CI)	*p*
North			
Total Period	2000–2017	−2.8 (−3.5; −2.2)	<0.001
Period 1	2000–2007	−4.5 (−4.7; −4.7)	<0.001
Period 2	2007–2013	−2.0 (−2.3; −1.7)	<0.001
Period 3	2013–2017	−1.2 (−1.6; −0.7)	<0.001
East			
Total Period	2000–2017	−4.3 (−4.5; −4.1)	<0.001
Period 1	2000–2004	−3.1 (−3.3; −3.0)	<0.001
Period 2	2004–2009	−5.0 (−5.2; −4.9)	<0.001
Period 3	2009–2015	−4.5 (−4.6; −4.4)	<0.001
Period 4	2015–2017	−0.2 (−0.3; 0.8)	0.370
Central			
Total Period	2000–2017	−2.7 (−2.8; −2.5)	<0.001
Period 1	2000–2002	−2.0 (−2.7; −1.2)	<0.001
Period 2	2002–2006	−3.9 (−4.2; −3.7)	<0.001
Period 3	2006–2011	−2.4 (−2.6; −2.4)	<0.001
Period 4	2011–2017	−2.0 (−2.0; −1.9)	<0.001
South			
Total Period	2000–2015	−4.8 (−5.0; −4.5)	<0.001
Period 1	2000–2004	−3.0 (−3.1.; −2.8)	<0.001
Period 2	2004–2007	−4.7 (−5.1; −4.3)	<0.001
Period 3	2007–2013	−6.0 (−6.1; −5.9)	<0.001
Period 4	2013–2017	−3.1 (−3.2; −3.0)	<0.001
West			
Total Period	2000–2015	−2.0 (−2.1; −1.8)	<0.001
Period 1	2000–2003	−1.5 (−1.8.; −1.3)	<0.001
Period 2	2003–2007	−3.5 (−3.7; −3.2)	<0.001
Period 3	2007–2014	−1.2 (−1.3; −1.2)	<0.001
Period 4	2014–2017	−1.9 (−2.1; −1.6)	<0.001

**Table 3:** Maternal Mortality Rates in the Organisation for Economic Cooperation and Development countries (OECD) 2017.

Country	MMR	Country	MMR
Colombia	83	Austria	5
Mexico	33	Belgium	5
Costa Rica	27	Ireland	5
Latvia	19	Japan	5
United States	19	Luxembourg	5
Turkey	17	Netherlands	5
Chile	13	Slovakia	5
Hungary	12	Switzerland	5
Republic of Korea	11	Denmark	4
Canada	10	Iceland	4
Estonia	9	Spain	4
New Zealand	9	Sweden	4
France	8	Czechia	3
Lithuania	8	Finland	3
Portugal	8	Greece	3
Germany	7	Israel	3
Slovenia	7	Italy	2
United Kingdom	7	Norway	2
Australia	6	Poland	2

Source of data: [58].

**Table 4:** Mother’s age at first birth, prenatal visits, and birth at a health facility by region.

Region	Mother’s Age at First Birth	4+ Prenatal Care Visits	Health Facility Births
Central	19.9	50.8%	71.6%
Eastern	19.7	55.5%	51%
Northern	23.5	72.3%	84.0%
Southern	20.6	60.1%	76.7%
Western	19.0	55.5%	51.4%
Total Africa	20.1	58.4%	64.5%

Source of data [61,100,101,102,103,104].

**Table 5:** Accessibility to the healthcare system in Africa by regions.

Indicator	Central	East	South	West
Accessibility ^†^	84.9%	91.7%	80.2%	97.9%
Sustainability follow-up in pregnancy ^‡^	82.5%	89.5	71.9%	86.6%
Sustainability follow-up in puerperium ^¥^	50.9%	58.6%	65.2%	17.1%

Source of data [115]. ^†^ Any ARV during pregnancy ^‡^ 3 ≥ ARV during pregnancy ^¥^ Any ARV in an infant after birth.

## Data Availability

UNICEF’s data are available on the internet at https://data.unicef.org/wp-content/uploads/2019/09/MMR-maternal-deaths-and-LTR_MMEIG-trends_2000–2017_Revised-2021.xlsx (accessed on 21 July 2022) [58].
